# Visual Analysis of Global Carbon Mitigation Research Based on Scientific Knowledge Graphs

**DOI:** 10.3390/ijerph19095766

**Published:** 2022-05-09

**Authors:** Na Su, Zhenbo Wang

**Affiliations:** 1 Key Laboratory of Regional Sustainable Development Modeling, Chinese Academy of Sciences, Beijing 100101, China; 18366186920@163.com; 2 College of Resources and Environment, University of Chinese Academy of Sciences, Beijing 100049, China

**Keywords:** scientific knowledge graphs, carbon mitigation, bibliometrics, hot spot analysis, trend analysis

## Abstract

Global temperature change is related to the destiny of all mankind, and carbon mitigation, as well as greenhouse gases control, are key points. In order to explore the basic knowledge, research hotspots and trends in global carbon mitigation research, this paper, based on 15,304 carbon mitigation articles from Web of Science, from 1991 to 2021, conducts spatio-temporal distribution, country distribution, research hotspots and cooperation network analysis, and draws frontier knowledge graphs of carbon mitigation by using CiteSpace, Gephi and other scientific knowledge mapping and literature analysis software. The key scholars, important literature, main contribution institutions and countries/regions in the field of carbon mitigation research were extracted, and the research basis and evolution law were displayed. The study found that: (1) The research on carbon mitigation is increasing every year, which can be roughly divided into four stages: germination, low-speed development, medium-speed development and high-speed development. (2) The spatial distribution of carbon mitigation research is unbalanced, mainly showing a characteristic of “one super and many strong” centered on the United States. (3) The research hotspots of carbon mitigation have gradually evolved from phenomenon analysis, policy guidance, method exploration to mechanism improvement. Further research might focus on ocean carbon sink, carbon trading and carbon-negative technology.

## 1. Introduction

Global temperature change is one of the most serious challenges facing human society in the 21st century, which is related to human survival and development [1]. The Intergovernmental Panel on Climate Change (IPCC) believes that the increase in the concentration of greenhouse gases, such as CO_2_, is the main factor driving global warming [2]. Although this view is still controversial in academia, carbon mitigation is still the focus of energy strategy development in various countries. The increasing harm caused by climate change has brought severe challenges to global economic development. How to effectively reduce carbon emissions has become one of the hotspots of international political, economic and academic research. However, the earth is an extremely complex dynamic system, and there are many factors affecting climate change, in addition to human factors, such as greenhouse gases produced by human activities. It also includes natural factors, such as solar activity and volcanic eruptions.

Although the source of carbon mitigation still remains controversial, at present, carbon reduction is not only highly valued by government departments, but also attracts the attention of many researchers. Garcia, Lewis and Griscom suggest that photosynthetic carbon capture by trees may be one of the most effective strategies to limit the rise of global CO_2_ concentration [2,3,4,5]. Asumadu and Keywan indicate that the adoption of clean and modern energy technologies is an effective means to reduce carbon emissions [6,7]. Woolf, Warnock and others believe that biochar is an important way to mitigate climate change through carbon sequestration [8,9]. Marshall, Katherine and Allison discuss the relationship between land use and carbon mitigation [10,11]. With the global emphasis on carbon mitigation, the relevant literature is becoming more and more abundant, mainly including climate change and agricultural carbon mitigation [12,13], the impact of land use change on carbon emissions [14,15], carbon emission reduction and climate policy [16], ecosystem services and carbon emission reduction [17,18], forest ecosystem and carbon emission reduction [19,20] and other related research progress.

To sum up, in addition to carbon mitigation methods, relevant research also involves agriculture, ecosystems, land use and other aspects. Particularly, research panorama, spatial distribution, regional disparities and hotspots are important for promoting carbon mitigation. In order to grasp the panorama of carbon mitigation research, regional disparities and other research status, this study, based on the global literature data of Web of Science (WoS) (https://www.webofscience.com/, accessed on 4 January 2022), uses dynamic network analysis and visual techniques and tools to analyze the research structure, research hotspots and future development trends of carbon mitigation, which provide an important reference for research in the future. The aim of this study is to analyze the intellectual structure, hotspots and trends of global carbon mitigation.

## 2. Materials and Methods

### 2.1. Data Sources

Global carbon mitigation research involves many disciplines, such as environmental science, energy fuels, environmental research, green sustainable development, meteorology, economics, ecology and so on. WoS is an important database for obtaining academic information worldwide. The Web of Science Core Collection (WoSCC) covers the world’s most influential research findings. Based on the WoSCC information retrieval platform, this study extracted the global carbon mitigation research literature with the theme of “carbon mitigation”, covering the period from 1991 to 2021, and retrieved a total of 18,314 articles. The data of English literature from the WoSCC database was retrieved on 4 January 2022 through a search by “TS” = (carbon mitigation) for all years of the collection span. After duplication screening with CiteSpace, a total of 18,314 articles were found, spanning from 1991 to 2021. After data normalizing and filtering (only articles in category, excluding repeated publication, non-officially published and other incomplete information), a total of 15,304 papers were retained as research samples for trend analysis of global carbon emissions research.

### 2.2. Research Methods

CiteSpace is an information visualization software developed by Professor Chen Chaomei, School of Computing and Informatics, Drexel University, USA, based on White and Griffith’s [21] author co-citation analysis theory and Kuhn’s [22] scientific structure evolution theory using Java language [23,24,25,26,27]. The software is mainly used to analyze and visualize the author co-citation network, generate knowledge concept graphs and knowledge clustering graphs, to help scholars explore research hotspots, frontiers and potential new trends [28].

CiteSpace has the most comprehensive functions to process and analyze the documents in WoS database, mainly including national/regional cooperation network, institutional cooperation network, author cooperation network and co-occurrence or co-word analysis based on references, keywords, subject headings, etc. This paper mainly analyzes the knowledge base, discipline structure and research frontier in the field of carbon mitigation through CiteSpace. The co-citation network composed of the cited literature can clearly reveal the knowledge base of a certain academic topic; keyword co-occurrence network can clearly show the disciplinary structure of a certain field; the citation groups and references cited by the citation groups can show the frontier characteristics of the research in this field. This paper analyzes the co-citation network, keyword co-occurrence network and emergence words of literature on carbon-mitigation-related research topics published from 1991 to 2021 in the WoSCC database to clarify the progress of carbon mitigation knowledge Graph.

The scientific knowledge graphs from CiteSpace analysis are mainly presented in the form of nodes and links. The size of nodes reflects the frequency of the analysis objects (such as countries/regions, institutions, authors, cited documents, journals, etc.); the thickness of links reflects the strength of cooperation, co-occurrence or co-citation; the color of nodes represents different years; the color of the line corresponds to the time of the first co-occurrence or co-citation of the node [29]. In CiteSpace, the clustering effect is usually reflected by Modularity (Q value) and Silhouette (S value). It is generally believed that Q > 0.3 represents significant clustering structure, S > 0.5 represents reasonable clustering, and S > 0.7 represents convincing clustering.

## 3. Results

### 3.1. Temporal and Spatial Distribution of Carbon Mitigation Research

#### 3.1.1. Temporal Distribution of Carbon Mitigation

**Figure 1:**
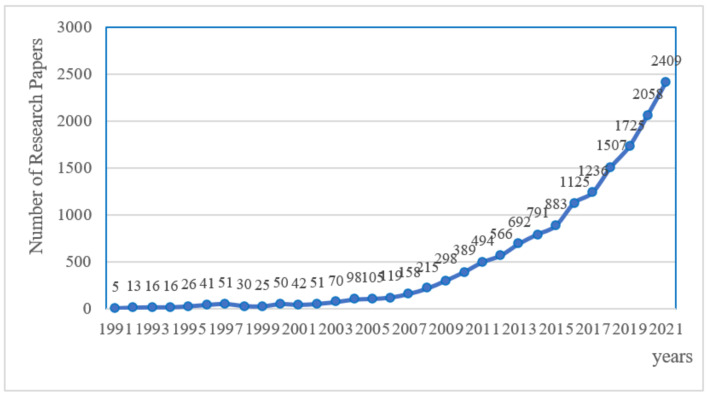
Annual Change Statistics on the Number of Research Papers Published in the WoSCC Database on Carbon Mitigation. Note: the data in the figure were obtained based on WOS summary and collation.

The interannual variation in the number of published papers in a certain field can reflect, to some extent, the attention that researcher pays to the field, which is helpful to the overall research and analysis of the development status and trend of a certain field. Figure 1 is the statistical chart of the annual change in the number of published articles on carbon mitigation in the WoSCC database from 1991 to 2021. It can be seen from the chart that the number of published articles on global carbon mitigation is increasing year on year; 1991–2002, the research on carbon mitigation is in the “embryonic” stage, and the number of papers published in this stage is relatively small. From 2002 to 2007, although the number of published articles increased compared with the previous stage, the growth rate was slower. From 2007 to 2016, the number of papers on carbon mitigation increased linearly, and the research on carbon mitigation was in a state of uniform development. The fourth comprehensive report from the IPCC [1] further promoted the research on carbon mitigation. By 2016, the Paris Agreement [30] was formally implemented. Carbon mitigation research has also been more widely considered by scholars. At this stage, the number of published papers is growing exponentially, and research is developing at a high speed.

#### 3.1.2. Country/Region Distribution of Carbon Mitigation Studies

**Figure 2:**
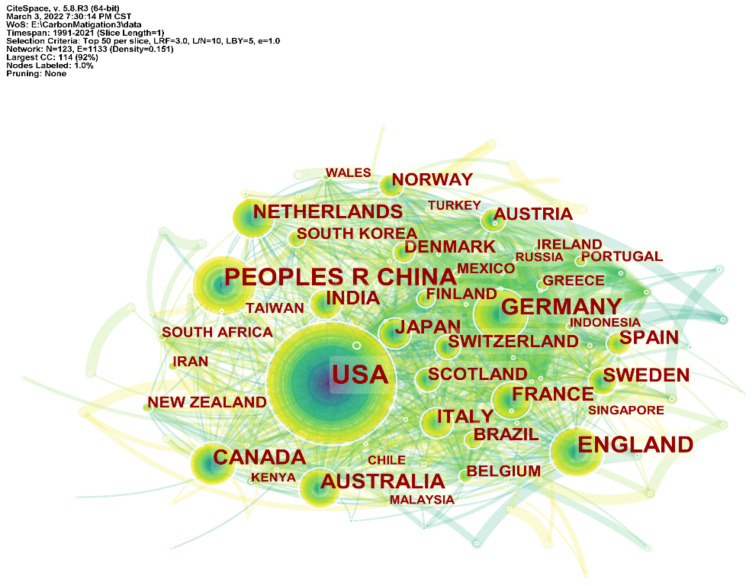
Map of National/Regional Cooperation Networks in the Field of Carbon Mitigation Research.

**Figure 3:**
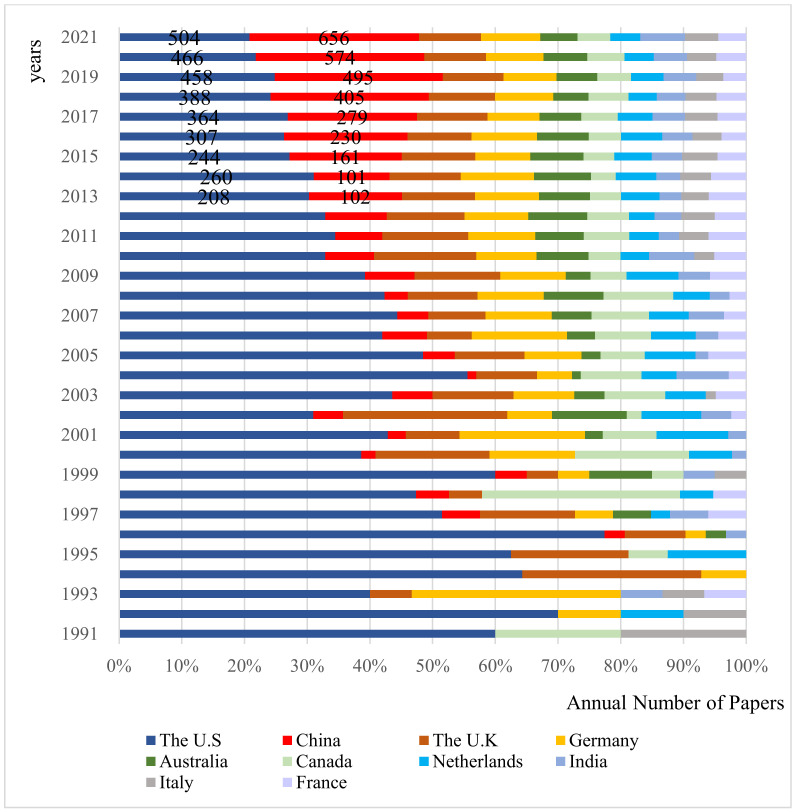
Annual Number of Papers Published by the Top Ten Countries/Regions in the Field of Carbon mitigation Research.

The number of publications in a country/region in a certain field usually represents the degree of activity in that field, which helps to analyze the relationship between countries/regions. In this paper, CiteSpace is used to map the national/regional cooperation network in the field of carbon mitigation (Figure 2), to analyze the cooperation network of major countries. Table 1 shows the top ten countries/regions, in terms of carbon mitigation, which are also the main research countries/regions in the field of carbon mitigation, and degree represents the number of cooperating countries/regions. Shown in Figure 3 are the annual statistics of the top ten countries/regions in the field of carbon mitigation. From 1991 to 2021, 167 countries/regions published articles related to carbon mitigation. From the analysis of Figure 2 and Table 1, the United States has always been in the leading position in the field of carbon mitigation, not only with the largest number of publications (4213), but also the highest cooperative network intensity (132), and the earliest countries/regions to participate in carbon mitigation research. They are followed by China (3180) and the United Kingdom (1686). Although the number of papers published in China is far more than that in the UK, the intensity of the cooperation network is slightly lower than that in developed countries, such as the UK The history of China’s participation in carbon mitigation research is also slightly shorter than that in developed countries, such as the UK and Germany. In addition, among the top ten countries/regions in terms of carbon mitigation, except for China and India, the rest are developed countries, which demonstrates that developed countries pay more attention to carbon mitigation research and make major contributions in this academic research field.

From the analysis of China’s nodes and statistical data, China’s scientific research participation in this field was relatively low in the early stage of carbon emission reduction research and the period of low-speed development. In recent years, as more and more Chinese scholars have joined carbon emission reduction research, China’s publication volume in this field has increased sharply. Since 2018, it has surpassed the United States to become the country with the highest number of publications in this field. It can be seen that China is paying more and more attention to the research on carbon mitigation.

### 3.2. Knowledge Base, Discipline Structure and Scient

Distribution of Research and Funding Institutions for Carbon Mitigation Research

**Figure 4:**
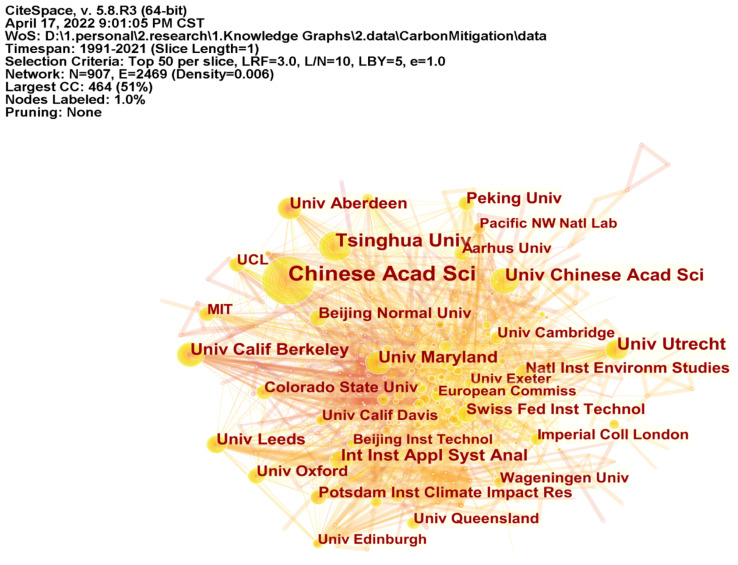
Cooperation Network of Institutions in the Field of Carbon Emission Reduction Research.

National scientific research institutions represent strategic scientific and technological strength, and funding institutions reflect the degree of attention. Figure 4 illustrates the distribution of the cooperation network between research institutions and funding institutions. Table 2 shows the top ten research institutions/funding institutions in carbon mitigation, and degree represents the number of cooperative institutions. It can be seen from Figure 4 and Table 2 that in the field of carbon mitigation, from 1991 to 2021, the Chinese Academy of Sciences has the largest number of publications (714), has cooperated with 132 institutions in the field of carbon mitigation, and has played an important role in promoting the development of research in this field. The main funding agency is the National Natural Science Foundation of China. The University of California System (534) and the USA. Department of Energy (521) ranked second and third in the number of publications. The main funding agencies are the National Science Foundation and the USA. Department of Energy. The Chinese Academy of Sciences and Tsinghua University are among the top ten research institutes in China. Other research institutes are from developed countries, which show a similar trend as the number of national publications.

### 3.3. Knowledge Base, Discipline Structure and Scientific Frontier of Scientific Knowledge Mapping Analysis of Carbon Emission Reduction Research

#### 3.3.1. Knowledge Base for Carbon Mitigation Research

**Figure 5:**
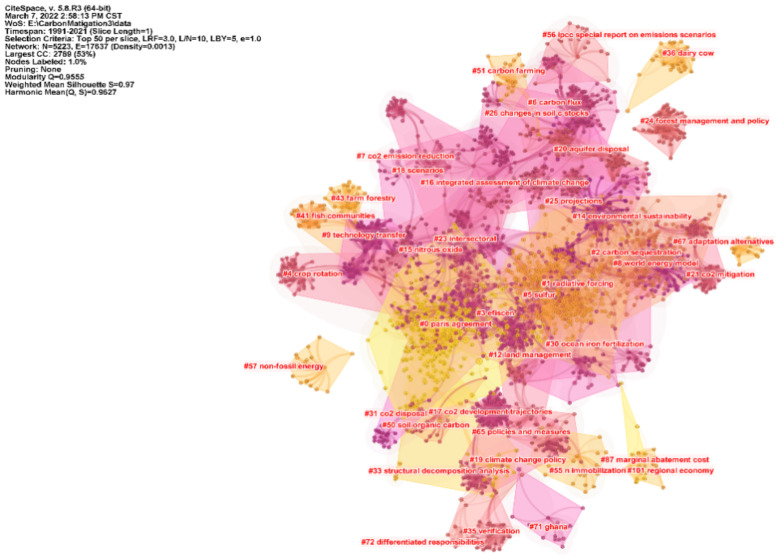
Knowledge Map of Keyword Clustering of Cited Literature in Carbon Emission Reduction Research.

**Figure 6:**
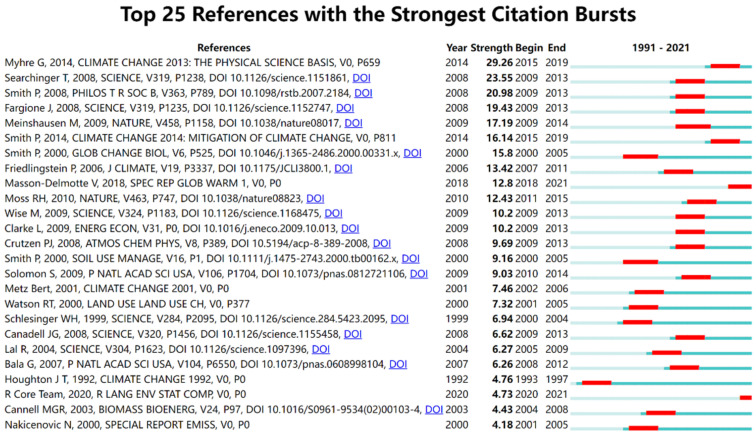
Top 25 Cited Studies on Carbon Emission Reduction Research. Note: the top 25 cited literature sources: (1) AR5 Climate Change 2013: The Physical Science Basis—IPCC (https://www.ipcc.ch/report/ar5/wg1/) accessed on 1 March 2022. (2) https://www.science.org/doi/10.1126/science.1151861 accessed on 1 March 2022. (3) https://royalsocietypublishing.org/doi/full/10.1098/rstb.2007.2184 accessed on 1 March 2022. (4) https://www.science.org/doi/10.1126/science.1152747 accessed on 1 March 2022. (5) Greenhouse-gas emission targets for limiting global warming to 2 °C|Nature (https://www.nature.com/articles/nature08017) accessed on 1 March 2022. (6) AR5 Climate Change 2014: Impacts, Adaptation, and Vulnerability—IPCC (https://www.ipcc.ch/report/ar5/wg2/) accessed on 1 March 2022. (7) https://onlinelibrary.wiley.com/doi/abs/10.1046/j.1365-2486.2000.00331.x accessed on 1 March 2022. (8) Climate–Carbon Cycle Feedback Analysis: Results from the C4MIP Model Intercomparison in: Journal of Climate Volume 19 Issue 14 (2006) (ametsoc.org) (https://journals.ametsoc.org/view/journals/clim/19/14/jcli3800.1.xml) accessed on 1 March 2022. (9) SR15_SPM_version_stand_alone_LR.pdf (ipcc.ch) (https://www.ipcc.ch/site/assets/uploads/sites/2/2018/07/SR15_SPM_version_stand_alone_LR.pdf) accessed on 1 March 2022. (10) The next generation of scenarios for climate change research and assessment | Nature (https://www.nature.com/articles/nature08823) accessed on 1 March 2022. (11) Implications of Limiting CO_2_ Concentrations for Land Use and Energy (https://www.science.org/doi/epdf/10.1126/science.1168475) accessed on 1 March 2022. (12) International climate policy architectures: Overview of the EMF 22 International Scenarios-Science Direct (https://www.sciencedirect.com/science/article/pii/S0140988309001960?via%3Dihub) accessed on 1 March 2022. (13) ACP-N2O release from agro-biofuel production negates global warming reduction by replacing fossil fuels (copernicus.org) (https://acp.copernicus.org/articles/8/389/2008/) accessed on 1 March 2022. (14) Meeting the UK’s climate change commitments: options for carbon mitigation on agricultural land-Smith-2000-Soil Use and Management-Wiley Online Library (https://bsssjournals.onlinelibrary.wiley.com/doi/10.1111/j.1475-2743.2000.tb00162.x) accessed on 1 March 2022. (15) Irreversible climate change due to carbon dioxide emissions | PNAS (https://www.pnas.org/doi/full/10.1073/pnas.0812721106) accessed on 1 March 2022. (16) [PDF] Climate change 2001: mitigation | Semantic Scholar (https://www.semanticscholar.org/paper/Climate-change-2001-%3A-mitigation-Metz-Davidson/225e2605932bc6fef128a57c2d15d2b92a40c3b5) accessed on 1 March 2022. (17) Land Use, Land-Use Change, and Forestry—IPCC (https://www.ipcc.ch/report/land-use-land-use-change-and-forestry/) accessed on 1 March 2022. (18) Carbon Sequestration in Soils (science.org) accessed on 1 March 2022. (19) Managing Forests for Climate Change Mitigation (science.org) accessed on 1 March 2022. (20) Soil Carbon Sequestration Impacts on Global Climate Change and Food Security (science.org). (21) Combined climate and carbon-cycle effects of large-scale deforestation|PNAS (https://www.pnas.org/doi/full/10.1073/pnas.0608998104) accessed on 1 March 2022. (22) ipcc_wg_I_1992_suppl_report_front_matters.pdf, (https://www.ipcc.ch/site/assets/uploads/2018/05/ipcc_wg_I_1992_suppl_report_front_matters.pdf) accessed on 1 March 2022. (23) R Core Team (2020) R A Language and Environment for Statistical Computing. R Foundation for Statistical Computing, Vienna-References-Scientific Research Publishing (scirp.org) (https://scirp.org/reference/referencespapers.aspx?referenceid=3000405) accessed on 1 March 2022. (24) Carbon sequestration and biomass energy offset: theoretical, potential and achievable capacities globally, in Europe and the UK-ScienceDirect (https://www.sciencedirect.com/science/article/abs/pii/S0961953402001034?via%3Dihub) accessed on 1 March 2022. (25) Nakicenovic, N., et al. (2000) Special Report on Emissions Scenarios. Cambridge University Press, Cambridge, 599 p.-References-Scientific Research Publishing (scirp.org), (https://www.scirp.org/reference/ReferencesPapers.aspx?ReferenceID=1354892) accessed 1 March 2022.

**Figure 7:**
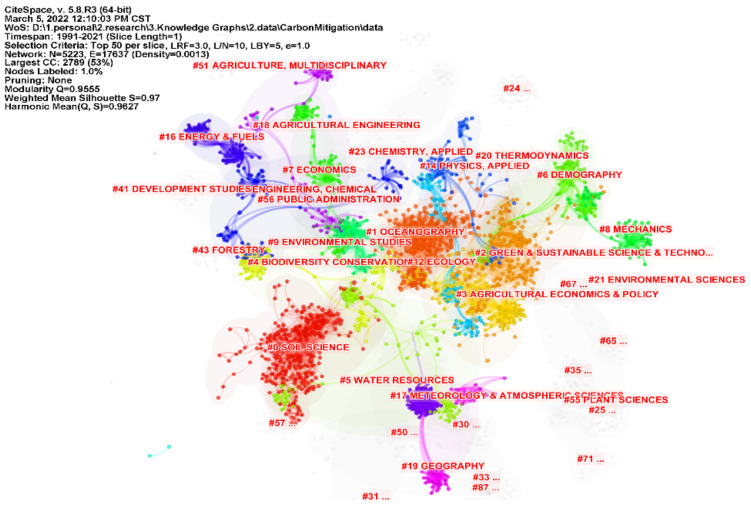
Knowledge Map of Cited Literature on Carbon Emission Reduction Research Based on Discipline Clustering.

The knowledge base of carbon emission research can be explored from the co-citation network constructed by the cited literature. Figure 5 shows the clustering knowledge map constructed by the keywords of the cited literature on carbon emission reduction, with Modularity Q of 0.9555 and Mean Silhouette of 0.97. The cluster structure is significant and convincing, with purple to yellow in the figure indicating changes in carbon mitigation study from 1991 to 2021. The smaller the rank of the cluster label is, the larger the cluster size is, and the more keywords it contains. Based on the keywords of the cited literature, the software automatically generated 41 clusters, which were re-classified according to the development context. The main contents of the initial study were Greenhouse gases, radiative forcing, carbon sequestration, etc. In the process of steady and uniform development, the research content has evolved to carbon sequestration, carbon capture, climate policy, biochar and so on. This is conducive to promoting the transformation and upgrading of high-energy-consuming industries, also triggering more in-depth research on biomass energy and other related research. With the implementation of international carbon emission reduction policies, the focus of research in various countries has shifted to the Paris Agreement, LMDI method, BECCS and CO_2_ Emissions. Figure 6 illustrates the 25 most influential cited studies. According to the citation intensity, the most influential one is *Climate Change 2013: The Physical Science Basis* [30], published in 2013. The implementation of policies plays a leading role in the development and promotion of a certain field. Secondly, in 2008, Searchinger, Heimlich and Houghton published an article on the impact of biofuel use on carbon emissions in the process of land use in Science, which was widely cited from 2009 to 2013, indicating that scholars attach importance to the relationship between rational land use and carbon emission reduction [31]. In 2008, Smith’s publication on greenhouse gas emission reduction in agriculture attracted much attention [32]. In the same year, Fargione discussed the carbon debt relationship between land reclamation and biofuels [33], which laid the foundation for the development of low-carbon economy. Figure 7 shows the basic clustering knowledge map of the cited literature on carbon emission reduction research. Soil Science, Agricultural Economics and Policy, Oceanography and Ecology are all closely related to carbon emission reduction research. The related research in these fields provides a basis for the knowledge system, technology realization and industrial transformation of carbon emission reduction.

#### 3.3.2. Discipline Structure of Carbon Emission Reduction Research

**Figure 8:**
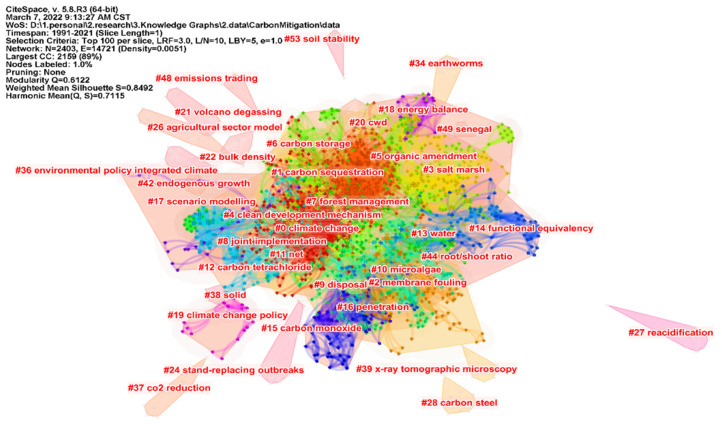
Knowledge Map of Keyword Clustering of Cited Literature in Carbon Emission Reduction Research.

The scientific knowledge map constructed by the key words of the cited literature can more intuitively reflect the discipline structure from the micro level. Figure 8 is the knowledge map of the keywords clustering of the cited literature on carbon mitigation research, with a Modularity Q value of 0.8492 and a Mean Silhouette value of 0.6122. The clustering structure is significant and convincing. It can be seen from the figure that the disciplinary structure of carbon mitigation is mainly divided into four aspects: defining concepts and carbon source exploration, policy formulation, technical methods and carbon mitigation measures. Carbon source exploration includes volcano degassing in addition to human factors. Policy making mainly includes environmental policy integrated with climate, emissions trading, climate change policy and joint implementation. The main technical methods are the agricultural sector model, reacidification, membrane fouling, organic amendment, etc. Carbon reduction measures include soil stability, clean development mechanism, forest management and earthworms.

#### 3.3.3. Scientific Frontier of Carbon Emission Reduction Research

**Figure 9:**
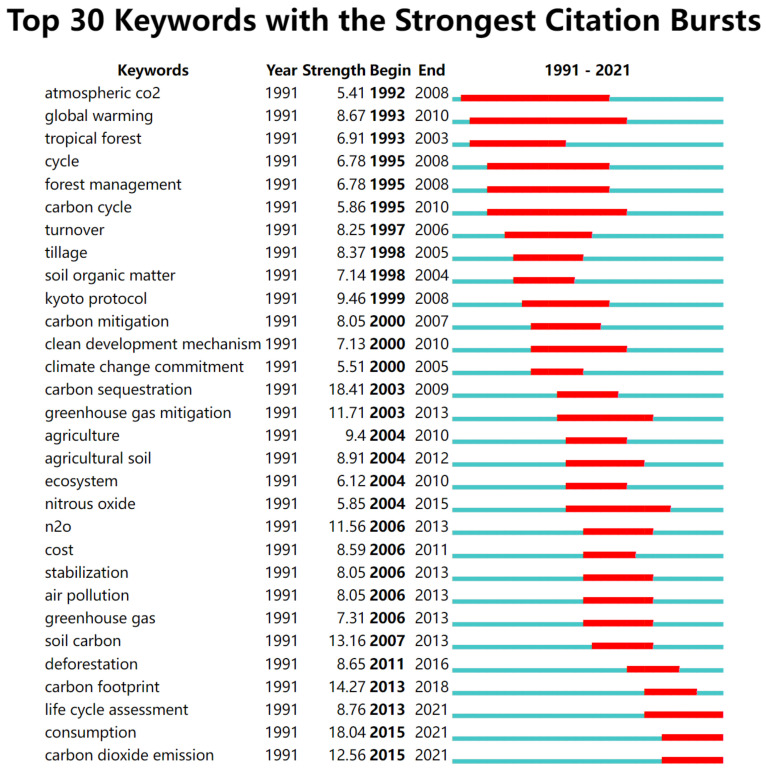
Key words highlighted in the first 30 citations of carbon emission reduction research.

In this paper, Burst Detection (Emergence Analysis) is used to analyze the keywords of the cited literature on carbon emission reduction, which can clearly show the scientific frontier and trend of carbon emission reduction. Figure 9 shows the keywords highlighted in the first 30 citations of carbon emission reduction research. As can be seen from the figure, 1991–2000 is the initial stage of carbon emission reduction research, with global warming, carbon cycle, forest management, and Kyoto Protocol representing the research frontier of this stage. This paper mainly discusses the carbon sources and carbon mitigation policies, among which global warming and atmospheric CO_2_ have been studied for the longest time, indicating that the relationship between global warming and atmospheric CO_2_ has been widely considered by scholars. From 2000 to 2012, the main emerging words in the reduction research are carbon sequestration, ecosystem, agriculture and clean development mechanism. This depicts that scholars have not only carried out long-term research on agricultural ecosystems and carbon sequestration, but have also gone deep into clean energy and other fields. From 2012 to 2021, the emerging words in this research field focus on carbon footprint, life cycle assessment, carbon dioxide emission and consumption, which reveals that carbon mitigation research has entered the phase of innovation system construction. Energy structure and its efficiency are the key factors affecting carbon emission reduction. Efficient renewable energy technology requires continuous innovation and accelerated docking with the carbon market, while technological innovation and market promotion are inseparable from strong support in policy and financial guarantees.

## 4. Discussion

### 4.1. Unbalanced Spatio-Temporal Distribution of Carbon Mitigation Research

From 1991 to 2021, the research literature related to carbon mitigation increased year on year, generally showing four stages of germination, low-speed development, uniform medium-speed development and high-speed development, and the number of papers published in various countries/regions is parallel to the overall trend. Among the developed countries, the United States, Britain, Germany, Australia, Canada, the Netherlands, Italy and France have made great contributions to carbon mitigation. Among the developing countries, besides China, India has also made great efforts to reduce carbon emissions. Although a latecomer in this field, China has surpassed the United States to become the country with the largest number of publications since 2008. From the analysis of the national/regional cooperation network, the United States has the most extensive cooperation in this field, despite the fact that China has published more articles than the United States. However, it is still essentially necessary to expand the network of international cooperation with wider and deeper impact. From the analysis of the institutional cooperation network, the Chinese Academy of Sciences has the largest number of publications and plays an important role among the global scientific research institutions.

Carbon mitigation is a public issue at the global level. In the context of the unbalanced spatial distribution of carbon mitigation research, it is necessary for all countries in the world to mobilize their efforts more actively to jointly respond to the challenges brought by carbon emissions, and to put forward requirements and measures for carbon mitigation based on the actual situations by different countries/regions.

### 4.2. Discipline Basis and Hotspots of Carbon Mitigation Research

In the process of carbon mitigation research, scholars, including Searchinger, Smith, Fargione and other scholars, have provided new branches and directions, laying the disciplinary foundation in this field. The research in Soil Science, Agricultural Economics and Policy, Oceanography, Ecology and other related fields provides a basis for the knowledge system, technology realization and industrial transformation of carbon mitigation. The discipline structure of carbon mitigation research can be divided into four aspects: concept and carbon source exploration, policy formulation, technical methods and carbon mitigation measures. It is worth noting that policy formulation plays a positive role in promoting the research in this field. The initial research on carbon mitigation focused on the status quo of global warming and the release of relevant international policies, such as the Kyoto Protocol and the Paris Agreement. Countries around the world have begun to explore carbon mitigation methods and the corresponding mechanisms from the aspects of carbon mitigation, such as renewable energy, clean development, land use and so on. At present, carbon mitigation has formed a mature institutional mechanism, which has been gradually improved in terms of methodology, such as LDMI and BECCS, and technical support, such as solid oxide electrolysis, life cycle assessment and carbon footprint.

The formulation of carbon mitigation policy has effectively promoted the relevant research, but the innovation of technical methods and the implementation of carbon mitigation measures cannot be separated from the support of the government and the guarantee of funds.

### 4.3. Future Development Trend of Carbon Mitigation Research

From the overall analysis of the emerging keywords, the current research hotspots include carbon capture and storage, biomass energy, clean energy and other new technologies, new materials related to emission mitigation technologies, and emission mitigation measures.

At present, scholars mostly focus on the research of land use, farmland ecosystem and other carbon sinks. However, the global ocean area accounts for about 70%, which has a huge potential for carbon sinks. With the development of economy and the shortage of land resources, marine carbon sinks will be more widely considered by scholars. Low-carbon-oriented economic development will provide a new development model for the global economy. The development, trading and related activities of low-carbon projects will be conducive to promoting low-carbon economic development. To achieve carbon neutrality, negative carbon emission technology will be conquered sometime in the future, which would make it possible to control carbon emissions and absorption.

Although carbon mitigation has attracted global attention and countries have responded positively and taken measures, it is still necessary to combine carbon mitigation with economic development in the future.

## 5. Conclusions

Based on the WoSCC data platform, 15,304 carbon mitigation research studies were retrieved, with the time, countries/regions and institutional distribution of the literature being studied using scientific knowledge graphs and bibliometrics. Meanwhile, the main research countries/regions and institutions in the world were also analyzed. Through analyzing the cited literature by the keywords, the prominent words and the prominent papers, this paper discusses the discipline foundation, research hotspots and future trends in this field. The following conclusions are drawn:

1. From 1991 to 2021, the research literature related to carbon mitigation increased year on year, and its development process can be roughly divided into four stages. From 1991 to 2002, it was in the embryonic stage, with a small number of articles and focusing on phenomenon analysis. From 2002 to 2007, although the number of published articles increased compared with the previous stage, the growth rate was slower. From 2007 to 2016, the number of published articles increased linearly and was in a state of uniform development. From 2016 to 2021, the number of published articles grew exponentially and is now in a high-speed development trend.
2. The spatial distribution of carbon mitigation research is unbalanced, mainly showing one super with multi-strong distribution. The United States is in a dominant position, in terms of the total number of papers, cooperation networks and research history, while China, Britain, Germany, Australia, Canada, the Netherlands and other countries are evenly matched in carbon mitigation research. Overall, developed countries have invested a lot in carbon mitigation research and made great contributions to this field. Except for China and India, other developing countries have a lower research input and contribution in this field. Institutional research also shows a similar trend. Carbon mitigation, as a public issue facing the world, needs to more actively mobilize countries around the world to jointly respond to the challenges posed by carbon emissions.
3. Carbon mitigation research has gradually evolved from phenomenon analysis, policy guidance, technical methods to emission mitigation measures, and the current research hotspots include carbon capture and storage, biomass energy, clean energy and other new technologies, new materials-related emission mitigation technologies, and emission mitigation measures. In the future, it may develop towards marine carbon sink, carbon trading, carbon-negative emission technology and so on. This paper reveals the development status of carbon mitigation research, and analyzes the hot research directions and development trends in this field, which can provide basic support for global carbon mitigation policy formulation and researchers in this field.

In this paper, only the English literature was analyzed, and there was a lack of analysis on carbon-mitigation-related research in the Chinese literature. In addition, there are differences in carbon mitigation studies in different regions, and the reasons for the length of the paper are not fully discussed. Due to the different parameter settings and data processing of CiteSpace software, the knowledge graphs obtained in this paper are slightly different, but the overall analysis and conclusions are reliable.

## Figures and Tables

**Table 1:** Top 10 Countries/Regions in the Field of Carbon mitigation Research.

Country	Number of Publications	Degree of Cooperative Network Intensity
The USA	4213	132
China	3180	104
The UK	1686	125
Germany	1479	112
Australia	1076	101
Canada	896	93
Netherlands	828	97
India	795	93
Italy	731	102

**Table 2:** Top Ten Research Institutions and Funding Institutions in the Field of Carbon Emission Reduction Research.

Research Institutions	Number of Publications	Degree of Cooperative Network Intensity	Funding Agencies	Number of Publications
Chinese Academy of Sciences, China	714	1148	National Natural Science Foundation of China, China	1806
University of California System, The USA	532	1220	European Commission	843
USA. Department of Energy, The USA	521	1039	UK Research and Innovation, The UK	764
Tsinghua University, China	336	450	National Science Foundation, The USA	561
USA. Department of Agriculture, The USA	319	639	Natural Environment Research Council, The UK	405
Centre National de la Recherche Scientifique, France	306	1099	United States Department of Energy, The USA	358
International Institute for Applied Systems Analysis, Austria	304	661	Fundamental Research Funds for the Central Universities, China	264
Potsdam Institute for Climate Impact Research, Germany	261	465	Engineering and Physical Sciences Research Council, The UK	248
Helmholtz Association, Germany	253	773	Conselho Nacional de Desenvolvimento Científico e Tecnológico, Brazil	193
Wageningen University & Research, Netherlands	246	660	United States Department of Agriculture, The USA	187

Note: The research institution is the institution where the author of the article is located and the funding institution is the source of funding for the article.

## References

[1] Fang J.Y., Zhu J.L., Wang S.P., Yue C., Shen H. (2011). Global warming, human-induced carbon emissions, and their uncertainties. Sci. China Earth Sci..

[2] Intergovernmental Panel on Climate Change (IPCC) (2018). An IPCC Special Report on the Impacts of Global Warming of 1.5 °C above Pre-Industrial Levels and Related Global Greenhouse Gas Emission Pathways.

[3] Bastin J.F., Finegold Y., Garcia C., Mollicone D., Rezende M., Routh D., Crowther T.W. (2019). The global tree restoration potential. Science.

[4] Griscom B.W., Adams J., Ellis P.W., Houghton R.A., Lomax G., Miteva D.A., Fargione J. (2017). Natural climate solutions. Proc. Natl. Acad. Sci. USA.

[5] Lewis S.L., Wheeler C.E., Mitchard E.T., Koch A. (2019). Regenerate natural forests to store carbon. Nature.

[6] Asumadu S.S., Vladimir S. (2019). Effect of foreign direct investments, economic development and energy consumption on greenhouse gas emissions in developing countries. Sci. Total Environ..

[7] Van Vuuren D.P., Stehfest E., Gernaat D.E., Doelman J.C., Van den Berg M., Harmsen M., de Boer H.S., Bouwman L.F., Daioglou V., Edelenbosch O.Y. (2017). The Shared Socioeconomic Pathways and their energy, land use, and greenhouse gas emissions implications: An overview. Glob. Environ. Change.

[8] Woolf D., Amonette J.E., Street-Perrott F.A., Lehmann J., Joseph S. (2010). Sustainable Biochar to Mitigate Global Climate Change. Nature.

[9] Warnock D.D., Lehmann J., Kuyper T.W., Rillig M.C. (2007). Mycorrhizal responses to biochar in soil—Concepts and mechanisms. Plant Soil.

[10] Wise M., Calvin K., Thomson A., Clarke L., Bond-Lamberty B., Sands R., Smith S.J., Janetos A., Edmonds J. (2009). Implications of limiting CO_2_ concentrations for land use and energy. Science.

[11] Quansheng G., Junhu D., Fanneng H., Yuan P., Mengmai W. (2008). Study on landuse, land cover change and carbon cycle in China over the past 300 years. Sci. China D Geosci..

[12] Smith P., Powlson D.S., Smith J.U., Falloon P., Coleman K. (2010). Meeting Europe’s climate change commitments: Quantitative estimates of the potential for carbon mitigation by agriculture. Glob. Change Biol..

[13] Roudier P., Sultan B., Quirion P., Berg A. (2011). The impact of future climate change on West African crop yields: What does the recent literature say?. Glob. Environ. Change.

[14] Pan G., Li L., Wu L., Zhang X. (2010). Storage and sequestration potential of topsoil organic carbon in China’s paddy soils. Glob. Change Biol..

[15] Smith P. (2008). Land use change and soil organic carbon dynamics. Nutr. Cycl. Agroecosyst..

[16] Pietzcker R.C., Longden T., Chen W., Fu S., Kriegler E., Kyle P., Luderer G. (2014). Long-term transport energy demand and climate policy: Alternative visions on transport decarbonization in energy-economy models. Energy.

[17] Chen W., Jing C., Cihlar J. (2000). An integrated terrestrial ecosystem carbon-budget model based on changes in disturbance, climate, and atmospheric chemistry. Ecol. Model..

[18] Liao C.Z., Luo Y., Fang C., Li B. (2010). Ecosystem Carbon Stock Influenced by Plantation Practice: Implications for Planting Forests as a Measure of Climate Change Mitigation RID B-8016-2010. PLoS ONE.

[19] Kim J., Kim T.K., Arritt R.W., Miller N.L. (2002). Impacts of Increased Atmospheric CO_2_ on the Hydroclimate of the Western United States. J. Clim..

[20] Wang G., Guan D., Peart M.R., Chen Y., Peng Y. (2013). Ecosystem carbon stocks of mangrove forest in Yingluo Bay, Guangdong Province of South China. For. Ecol. Manag..

[21] Howard D., White H.D., Griffith B.C. (1981). Author cocitation: A literature measure of intellectual structure. J. Am. Soc. Inf. Sci..

[22] Kuhn T.S. (1962). The Structure of Scientific Revolutions. Phys. Today.

[23] Chen C. (2017). Science Mapping: A Systematic Review of the Literature. J. Data Inf. Sci..

[24] Chen C., Hu Z., Liu S., Tseng H. (2013). Emerging trends in regenerative medicine: A scientometric analysis in Cite Space. Expert Opin. Biol. Ther..

[25] Chen C. (2006). Cite Space II: Detecting and visualizing emerging trends and transient patterns in scientific literature. J. Am. Soc. Inf. Sci. Technol..

[26] Chen C., Morris S. Visualizing evolving networks: Minimum spanning trees versus Pathfinder networks. Proceedings of the IEEE Symposium on Information Visualization 2003.

[27] Chen C. (2013). System and Method for Automatically Generating Systematic Reviews of a Scientific Field. U.S. Patent.

[28] Weichen J., Wenguang L., Mingmei Y. (2020). Using CiteSpace to analyze Chinese journal publications: An optimized research paradigm. Distance Educ. China.

[29] Chen Y., Chen C.M., Liu Z.Y., Hu Z.G., Wang X.W. (2015). The methodology function of CiteSpace mapping knowledge domains. Stud. Sci. Sci..

[30] Ofipcc W. (2013). Climate Change 2013: The Physical Science Basis. Contrib. Work..

[31] Searchinger T., Heimlich R., Houghton R.A., Dong F., Elobeid A., Fabiosa J., Yu T.H. (2008). Use of USA. Croplands for Biofuels Increases Greenhouse Gases through Emissions from Land-Use Change. Staff. Gen. Res. Pap. Arch..

[32] Smith P., Martino D., Cai Z., Gwary D., Janzen H., Kumar P., Smith J. (2008). Greenhouse gas mitigation in agriculture. Philos. Trans. R. Soc. B Biol. Sci..

[33] Fargione J., Hill J., Tilman D., Polasky S., Hawthorne P. (2007). Land clearing and the biofuel carbon debt. Science.

